# Recurrent PET FDG Uptake after Sequential Chemotherapy and Radiation Therapy for DLBCL of the Tibia: A Case Report and Review of the Literature

**DOI:** 10.1155/2011/163472

**Published:** 2011-10-19

**Authors:** Edward F. Miles, Luke Balsamo, David B. Turton, William Graf

**Affiliations:** ^1^Division of Radiation Oncology, Department of Radiology, Naval Medical Center Portsmouth, 620 John Paul Jones Circle, Portsmouth, VA 23708, USA; ^2^Division of Orthopedics, Department of Surgery, Naval Medical Center Portsmouth, 620 John Paul Jones Circle, Portsmouth, VA 23708, USA; ^3^Division of Nuclear Medicine, Department of Radiology, Naval Medical Center Portsmouth, 620 John Paul Jones Circle, Portsmouth, VA 23708, USA; ^4^Division of Interventional Radiology, Department of Radiology, Naval Medical Center Portsmouth, 620 John Paul Jones Circle, Portsmouth, VA 23708, USA

## Abstract

The aim of this paper is to report on the challenges associated with identifying disease recurrence following combined modality therapy (CMT) for primary lymphoma of the tibia in which an intramedullary nail has been placed. A patient with primary bone lymphoma (PBL) was treated with CMT (chemotherapy and radiation therapy). After a complete response, he has been followed for eighteen months by physical exam and radiographic imaging. Despite persistent increased tracer accumulation at the original site, he has no proven recurrence. Literature review showed a small number of retrospective, single institution reviews detailing clinical experience and expected outcome in patients treated with PBL limited to one bony site of disease. PBL presents a treatment challenge, particularly when a weight-bearing long bone is diffusely involved and followup is complicated after placement of stabilizing hardware. Close coordination of the oncology team and diagnostic radiology is required to ensure optimal outcome.

## 1. Introduction

Primary bone lymphoma (PBL) is an uncommon disease that accounts for approximately 3% of all primary bone malignancies and 5% of all extranodal non-Hodgkin's lymphoma cases [1]. The most common histologic subtype is diffuse large B-cell lymphoma (DLBCL), with a reported frequency that ranges from 66.3% of patients with PBL in a recent analysis of the Surveillance, Epidemiology, and End Results (SEER) database [2] to as high as 80–91% in other retrospective reviews [3–5]. The most common presenting symptom is bone pain, followed by pathologic fracture, palpable mass, and systemic “B” symptoms (fever, weight loss, and night sweats) [3, 5]. PBL is staged using the Ann Arbor classification that was originally developed for staging Hodgkin's disease [6]. However, when outcomes were reviewed for patients with aggressive (intermediate or high grade) non-Hodgkin's lymphoma, the Ann Arbor staging system could not distinguish between patients with favorable versus unfavorable prognoses [7]. As a result, the International Prognostic Index (IPI) [8] was developed to predict long-term survival in patients with aggressive non-Hodgkin's lymphoma. The IPI classifies patients into one of four risk categories based on age, serum lactate dehydrogenase (LDH), performance status, tumor stage, and number of involved extranodal sites [8]. Potential treatment options based on the stage and IPI score are R-CHOP (rituximab plus cyclophosphamide, doxorubicin, vincristine, and prednisone) for 3 cycles plus involved field radiation therapy (IFRT) or R-CHOP for 6 to 8 cycles plus or minus IFRT [9]. Two recent randomized trials of systemic chemotherapy options for patients with DLBCL have specified adjuvant radiation therapy to sites of bulky or extranodal disease (RICOVER-60 and MinT) after the completion of chemotherapy [10, 11].

## 2. Materials and Methods

Written informed consent was obtained from the subject who has approved this document for print, electronic publication, and reprinting in foreign editions. He has been given the opportunity to see this paper in its entirety.

The patient is a 49-year-old male who presented with left leg pain along the lateral calf that started after running. He was initially diagnosed with shin splints and managed conservatively with physical therapy for two months but his symptoms did not improve. After failure of conservative therapy, he was referred for further workup. A bone scan of his lower extremities was consistent with a stress fracture of the left tibia. Treatment with steroids improved his pain temporarily, but the pain returned after several weeks and became progressively worse. An MRI of his left lower extremity demonstrated scattered small lucencies along the midtibial diaphysis with associated cortical thickening and periosteal reaction but no soft tissue mass. His blood work showed an ESR of 17 mm/hr, a CRP of 10.6 mg/L, an LDH of 128 units/L, and a white blood cell count of 4.8 × 10^3^/*μ*L. An open biopsy of the left tibial bone was consistent with chronic inflammation only, with no evidence of malignancy or infection.

**Figure 1:**
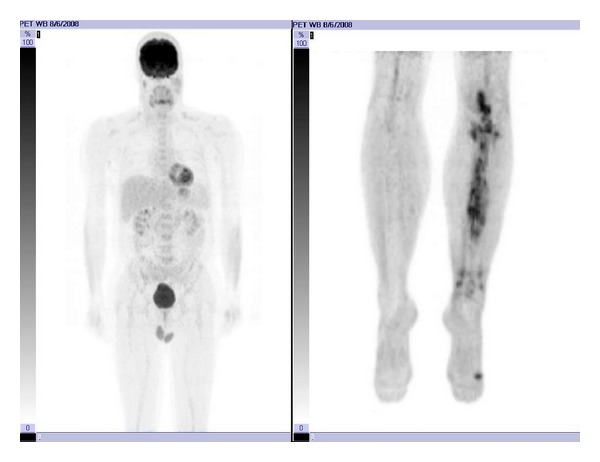
Pretreatment planar PET/CT image.

**Figure 2:**
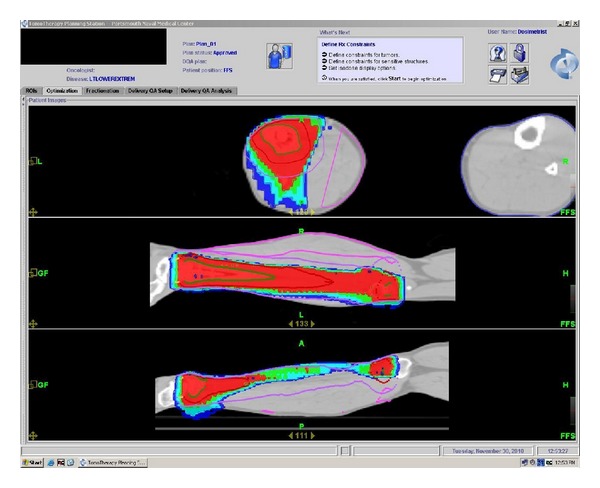
Tomotherapy planning study images demonstrating dose distribution and relative sparing of posterior compartment.

**Figure 3:**
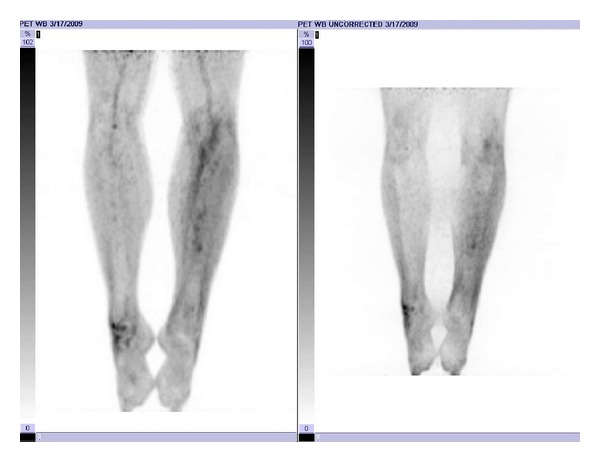
Postchemotherapy and radiotherapy planar PET/CT image.

After consultation with infectious disease, the patient was treated with antibiotics for what was thought to be osteomyelitis. This initially relieved his symptoms. After he completed his antibiotic course, his pain and swelling returned. A second open biopsy was performed by an orthopedic oncologist during which an area of spongy bone was resected and identified as germinal center DLBCL. An intramedullary (IM) nail with cortical/cancellous bone allograft was placed following the biopsy to prevent pathologic fracture due to the large amount of bone removed. Staging studies done prior to placement of the IM nail, including a skeletal survey and CT of the chest, abdomen and pelvis, were all negative for any additional lesions. A bone scan, [18F] fluoro-2-deoxy-d-glucose (FDG) positron emission tomography (PET)/CT, and bone marrow biopsy were completed and were also negative for involvement outside the original left tibial lesion, and he was staged as IAE DLBCL [6]. Activity in the patellar region of the initial PET/CT scan was thought to be related to the surgical intervention (Figure 1). Based on the patient's stage and IPI score of 0, he would have a predicted 5-year survival of between 83 and 90% [8, 12], and his recommended treatment would be R-CHOP for 3 cycles followed by IFRT [9]. Following the third cycle of R-CHOP, the patient was judged to have a complete response based on a repeat PET/CT. The patient elected to continue with the treatment course as recommended by the National Comprehensive Cancer Network (NCCN) clinical guidelines [9] and presented to Radiation Oncology for consideration of consolidation radiation therapy. A CT planning study was completed with the patient in the supine position with an alpha cradle for positioning and placement. The CT simulation was fused with his initial PET/CT scan and used to plan his radiation. We elected to treat the entire length of the tibia as the involved field, due to the diffuse nature of the disease on the pretreatment imaging (Figure 1) and the apparent involvement of the marrow space. Treating the entire length of the left tibia was problematic in that either an AP/PA or opposed lateral field arrangement would invariably treat the limb circumferentially with an associated long-term risk of limb edema. To limit this risk, IMRT was utilized in order to preferentially spare the posterior compartment of his left lower leg. He was treated via Tomotherapy to the entire tibia with 200 cGy fractions to a total of 4400 cGy (Figure 2) and was able to complete his radiation therapy without any treatment breaks. A posttreatment PET/CT scan showed no residual activity (Figure 3).

After completion of his radiation treatments, the plan was to continue following the patient with serial labs and imaging to monitor for disease recurrence. A subsequent PET/CT scan, three months after completion of radiation therapy, showed no evidence of focal activity at the site of original disease or at any distant site. Serum LDH at this time was also normal at 324 units/L. An MRI showed no evidence of residual disease; however, the exam was limited by metal artifact from the IM nail. The CT portion of the PET/CT studies also had significant artifact and in conjunction with Radiology, the decision was made to continue following the patient with serial PET/CT scans only. 

**Figure 4:**
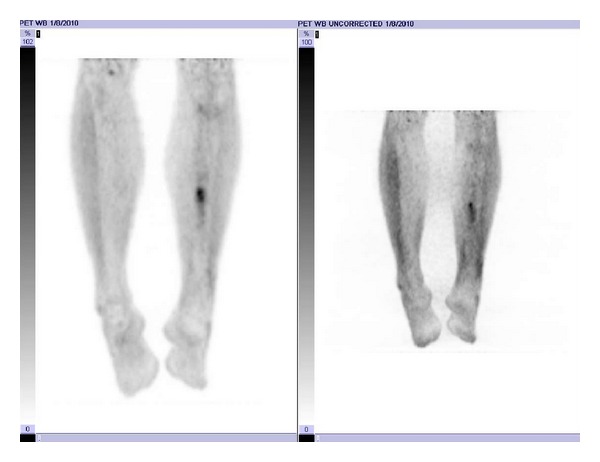
Posttreatment planar PET/CT image demonstrating recurrence of PET-avidity at site of original disease.

A subsequent PET/CT scan, nine months after completion of radiation therapy, showed increasing metabolic activity at the previous site of disease (Figure 4). A CT guided needle biopsy of the area was done initially that was nondiagnostic. An open biopsy was then performed, again, by an orthopedic oncologist, that also showed no evidence of malignancy. A CT scan of the left lower extremity taken after the open biopsy confirmed that the focus of increased 18F FDG accumulation was appropriately biopsied and that the biopsy area was adequate. Due to the two negative biopsies, the oncology team recommended followup after three months with physical exam and repeat PET/CT. 

**Figure 5:**
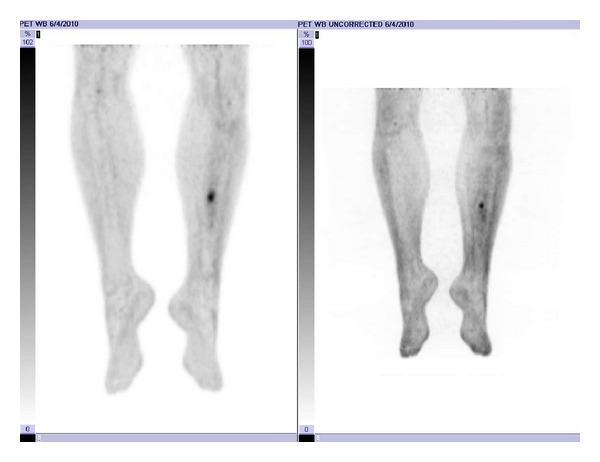
Postrebiopsy planar PET/CT image demonstrating no evidence of contiguous spread nor systemic recurrence.

His next PET/CT scan, 13 months following radiation therapy, again showed the area of increased glucose metabolism at the medial aspect of the left midtibial shaft with subtle increase in size and metabolic activity with standardized uptake value (SUV) of 3.3 compared to 3.1 previously. LDH at this time continued to be within normal limits. A second open biopsy of the left tibia was performed that showed only viable bone and necrotic inflammatory debris with no evidence of malignancy. Repeat PET/CT scans, labs, and physical exams, most recently nearly two years after completion of combined modality therapy (CMT), have continued to show only the one area of elevated 18F FDG activity near his original site of disease with no evidence of distant failure (Figure 5). The level of elevated activity has been decreasing slowly and his LDH remains within normal limits.

## 3. Results and Discussion

Due to the scarcity of patients with PBL, no randomized trials exist to evaluate treatment options. Radiation therapy was the standard of care for PBL beginning in the 1960s [13]. Radiation alone provided good local control; however, as many as 50% of patients treated with radiation alone failed systemically with regional or distant metastases [13, 14]. Several retrospective reviews published within the last five years have demonstrated improved outcomes with the use of combined treatment of multiple agent chemotherapy and radiotherapy [3–5, 15, 16]. 

Beal et al. reported on a single institution experience and identified 82 patients diagnosed and treated with PBL between 1963 and 2003 [3]. Of the 82 patients, 46 received a combination of chemotherapy and radiation, 11 received radiation alone, and 24 received chemotherapy alone. Analysis of outcomes showed a significant improvement with CMT. Chemotherapy was given in the form of CHOP or R-CHOP. For the patients who received radiation, either alone or following chemotherapy, the median dose was 4400 cGy. The reported 5-year overall survival for patients treated with combined modality versus single modality treatment was 95% versus 78%. 

In a retrospective review, Dubey et al. reported on their experience treating 45 patients with stage IE and IIE PBL between 1967 and 1992 [17]. Of the 45 patients, 36 were treated with CMT, 5 were treated with radiation alone, and 4 were treated with chemotherapy alone. Patients who received radiation were treated with doses between 4000 and 6000 cGy. Among the patients who received CMT, no local failures were reported with doses of 4400–4600 cGy, and the writers concluded that doses in the range of 4600 cGy allow for optimal local control with acceptable rates of complication. 

Christie et al. recently reported on a prospective study to evaluate limited chemotherapy and radiotherapy for PBL [18]. Treatment included 3 cycles of CHOP followed by radiation to a dose of 4500 cGy using a shrinking field technique. The five-year local control rate was 72% with 9 local failures among the 31 patients included in the study. The number of local failures led them to conclude that higher doses of radiotherapy (4000–4500 cGy) should be used to treat PBL than those usually prescribed for lymphoma in other tissues. 

Baar et al. reported on a series of patients treated for PBL at a single institution and described the difficulty with assessment of treatment response based on persistent abnormalities on imaging after treatment [19]. Those abnormalities are thought to represent bone remodeling or fibrosis. Placement of hardware within the bone to stabilize it can further complicate assessment of CT scans and MRIs via the artifact created by the metal rod.

The Rare Cancer Network recently reported on a multicenter retrospective review of 116 patients with stages I and II PBL that looked at prognostic factors and patient outcomes [20]. Important prognostic factors were identified as age, IPI score, complete response, chemotherapy, number of chemotherapy cycles, and radiation dose. Based on their analyses, PBL treated with CMT that included radiation doses of greater than 4000 cGy and at least six cycles of chemotherapy was associated with improved outcomes. The local control rate was 92% with median followup of 41 months. 

The patient in this report had a complete response to chemotherapy. Three months after completion of his radiation, the PET/CT scan continued to show no evidence of local recurrence or distant failure. However, an MRI completed following radiation therapy demonstrated the challenges associated with monitoring for disease recurrence in cases where hardware must be placed to prevent pathologic fractures. As previously noted, the posttreatment MRI and CT scans both demonstrated significant artifacts caused by the IM titanium rod that interfered with interpretation of changes and limited their usefulness as surveillance tools. Therefore, the decision was made to limit surveillance imaging to PET/CT scans. 

Nine months after completing his chemotherapy and radiation treatments, a PET/CT scan began to show a focal lytic lesion in the left midtibia cortex with increased 18F FDG uptake near the site of original disease. Initially, both a CT guided needle biopsy and open biopsy were both negative for disease recurrence. Serial PET/CT scans since that time have shown gradual increase in the size and activity of the lesion. However, the lesion was not definitive for recurrence and could represent posttreatment changes in the bone. 18F FDG accumulation in necrotic bone has been previously described [21, 22]. A third biopsy, this time another open biopsy, again failed to show any malignant cells. Following the third negative biopsy, the orthopedic surgeon recommended to the oncology team against any additional sampling to avoid further weakening of the bone.

In the case of biopsy-proven disease relapse or distant failure, the next recommended treatment would be high-dose chemotherapy with autologous stem cell transplant. However, in the absence of definitive evidence of local or distant failure and with the repeated negative biopsies, the current plan is to continue surveillance and repeat PET/CT every 3 months.

## 4. Conclusion

The optimal treatment of PBL appears to be CMT, with systemic chemotherapy followed by IFRT to a dose in the range of 4400 cGy. Treatment of the entire length of a bone is difficult with traditional field arrangements, and IMRT is useful to limit the risk of distal extremity lymphedema. Assessment of local disease control is complicated by bone remodeling and the potential for artifact due to stabilizing hardware. Careful coordination with all members of the oncology team, nuclear medicine, and radiology is essential for optimal outcomes.
